# Evaluation of systemic inflammatory and nutritional indexes in locally advanced gastric cancer treated with adjuvant chemoradiotherapy after D2 dissection

**DOI:** 10.3389/fonc.2022.1040495

**Published:** 2022-10-27

**Authors:** Shu-Bei Wang, Jia-Yi Chen, Cheng Xu, Wei-Guo Cao, Rong Cai, Lu Cao, Gang Cai

**Affiliations:** Department of Radiation Oncology, Ruijin Hospital, Shanghai Jiaotong University School of Medicine, Shanghai, China

**Keywords:** chemoradiotherapy, locally advanced gastric cancer, neutrophil-lymphocyte ratio (NLR), prognosis, para-aortic lymph nodes

## Abstract

**Background:**

Many studies have shown that the peripheral blood inflammatory index and nutritional index, such as the platelet lymphocyte ratio (PLR), neutrophil lymphocyte ratio (NLR), lymphocyte monocyte ratio (LMR), systemic inflammation response index (SIRI), pan-immune-inflammation value (PIV), systemic immune-inflammation index (SII), and prognostic nutrition index (PNI), are independent prognostic factors for tumors. The present study aimed to investigate the prognostic role of these peripheral blood indexes before treatment in locally advanced gastric cancer (LAGC) treated with adjuvant chemoradiotherapy after D2 dissection.

**Methods:**

A total of 89 patients with LAGC who underwent D2 gastrectomy and adjuvant chemoradiotherapy at our hospital from 2010–2018 were eligible. Systemic inflammatory indicators before treatment were evaluated. Receiver operating characteristic curve (ROC), Kaplan–Meier analysis, and Cox regression were utilized for prognosis evaluation.

**Results:**

The median follow-up time was 29.1 (4.1–115.8) months. The overall survival at 3 years (OS) and the disease-free survival (DFS) were 78.9% and 59.1%, respectively. According to the ROC curve for 3-year DFS, the best cut-off values of pre-treatment NLR, PLR, LMR, SII, SIRI, PIV and PNI were 1.7, 109.3, 2.9, 369.2, 0.58, 218.7, and 48, respectively. Multivariate Cox regression analysis showed that NLR was an independent prognostic factor for DFS (HR 2.991, 95%CI 1.085–8.248, *P =* 0.034). Kaplan-Meier analysis showed that a higher NLR (>1.70) was significantly associated with a poorer OS (3-year OS: 68.8% vs 92.9%, *P =* 0.045) and DFS (3-year DFS: 47.5% vs 80.9%, *P =* 0.005). In terms of the free locoregional recurrence rate (LRR), the prognosis of patients with high NLR was also significantly worse than those with low NLR (70.2% vs 96.0%, *P =* 0.017). Paraaortic lymph nodes were the most common site of LRR (7/14 patients). The seven cases of paraaortic lymph node metastasis occurred in patients with high NLR.

**Conclusions:**

In our retrospective analysis, we found that pretreatment NLR could serve as a prognostic factor for survival in LAGC treated with adjuvant chemoradiotherapy after D2 dissection, especially for the prediction of LRR and paraaortic lymph node metastasis. Prospective studies are needed to confirm our findings.

## Introduction

Gastric cancer is one of the leading causes of cancer mortality in China and worldwide (1, 2). Even with curative resection, 5-year survival in patients with locally advanced gastric cancer (LAGC) remains at 30–50% (3). Therefore, researchers have turned their attention to adjuvant and neoadjuvant treatment to improve both overall survival (OS) and quality of life in patients with LAGC (4).

According to early studies, more than 80% of patients who died from gastric cancer presented locoregional recurrence (LRR) (5), which led to the introduction of radiotherapy. The positive impact of adjuvant and neoadjuvant therapies on survival in patients with resectable LAGC has become increasingly clear. The INT 0116 trial (6, 7), which demonstrated a survival benefit from postoperative chemoradiotherapy, was compromised by the fact that >50 percent of the enrolled patients had an insufficient (less than D1) lymph node dissection, suggesting that adjuvant chemoradiotherapy could be primarily compensating for suboptimal lymph node surgery. As one of the largest trials for D2 lymph node dissection patients, the Adjuvant Chemoradiation Therapy in Stomach Cancer (ARTIST) trial (8) failed to show that the addition of radiotherapy to chemotherapy has a significant overall survival benefit. Only subgroup analyses showed that chemoradiotherapy significantly improved disease-free survival (DFS) in patients with gastric cancer-node positive disease. However, in the successor trial ARTIST 2 (9), the addition of radiation therapy to chemotherapy did not appear to provide additional benefit in patients with node-positive disease. Through the above studies, we found that the clinical benefits brought by postoperative radiotherapy are not universal for all patients after D2 lymphadenectomy.

Therefore, it is essential to find effective predictors and to accurately stratify patients with gastric cancer who may benefit from chemoradiotherapy. Inflammation plays a key role in the occurrence and development of tumors. Recently, several new indicators have been identified as independent prognostic markers for tumor outcome, such as the platelet lymphocyte ratio (PLR) (10), neutrophil lymphocyte ratio (NLR) (10–13), systemic immune inflammation index (SII) (14, 15), lymphocyte monocyte ratio (LMR) (16), systemic inflammation response index (SIRI) (17, 18), pan-immune-inflammation value (PIV) (19, 20), and prognostic nutrition index (PNI) (21, 22). Although some studies (12, 21) have also suggested that nutritional and inflammatory indexes are closely related to the prognosis of gastric cancer, the role of these indexes in gastric cancer treated with D2 dissection and chemoradiotherapy remains undetermined. Therefore, we conducted this study to evaluate the prognostic value of peripheral blood inflammatory and nutritional indicators in LAGC treated with adjuvant chemoradiotherapy following D2 dissection.

## Materials and methods

### Patient characteristics

The medical records of 89 patients with gastric or gastroesophageal junction adenocarcinoma who underwent radical D2 gastrectomy and adjuvant chemoradiotherapy at our hospital between January 2010 and December 2018, were retrospectively reviewed. The inclusion criteria were as follows: patients diagnosed with adenocarcinoma; stage IB-IIIC according to the eighth edition of the American Joint Committee on Cancer (AJCC) 8th edition (23); R0 and D2 was confirmed by postoperative pathology; presence of positive pathological lymph nodes; and received adjuvant chemoradiotherapy after the operation. This study was approved by the Institutional Medicine Review Board and a waiver for patient consent was obtained.

### Adjuvant chemotherapy and chemoradiotherapy

All patients received 6 (range 4–8) cycles of treatment regimens involving fluoropyrimidine after surgery. Concurrent chemotherapy regimens included tegafur or capecitabine (24).

All patients received 6MV linear accelerator radiotherapy with a total tumor dose of 41.4-54 Gy (1.8-2 Gy/time, 5 times/week). The tumor bed area, the anastomotic site, and the regional drainage lymph nodes (including paraaortic nodes) were defined as the clinical target volume (CTV). Radiotherapy plans were confirmed by senior radiotherapy clinicians. Before treatment, treatment fields, radiation dosimetry, surgical and pathological information, and preoperative imaging were checked.

### Data collection

All medical and surgical records were retrospectively reviewed, including baseline neutrophil, lymphocytes, monocytes, platelets, and albumin (g/L) levels, depth of tumor infiltration, number of positive lymph nodes, chemoradiotherapy, recurrence, and survival information. Recurrence of the tumor bed, anastomotic stoma, duodenal stump, gastric remnant, and regional lymph nodes were defined as LRR. Peritoneal dissemination referred to the recurrence that occurred in the peritoneum. All recurrences at distant sites were recorded as distant metastases (excluding the peritoneum) (25).

### Data definition

Laboratory examinations were performed before treatment. The calculation formula of each indicator of inflammatory markers was as follows: NLR = neutrophil count/lymphocyte count (21); PLR = platelet count/lymphocyte count; LMR = lymphocyte count/monocyte count (26); SII = platelet count × neutrophil count/lymphocyte count (27); SIRI = neutrophil count × monocyte count/lymphocyte count (28); PIV = neutrophil count × platelet count × monocyte count/lymphocyte count (19). PNI = serum albumin value + 5 × lymphocyte count (21).

### Statistical analysis

All statistical analyses were conducted with SPSS 26 software (IBM Corp., Armonk, NY, USA). Continuous variables were described using median and range, and categorical variables were presented as frequency and percentage. The receiver operating characteristic (ROC) curve was applied to determine the optimal cut-off value of these inflammatory markers based on the maximum Youden index. ROC curves were created by plotting the sensitivity against (1-specificity) for each parameter. The optimal cutoff value represented the maximum Youden index (sensitivity + specificity − 1). Prognostic factors for DFS were investigated using Cox regression analyses. Factors with a *p*-value <0.05 in the univariate analysis were then entered in the multivariate analysis, to identify independent prognostic factors. Survival curves were plotted using the Kaplan–Meier method and were compared using the logarithmic rank test. A *P*-value less than 0.05 was considered statistically significant.

## Results

### Patient characteristics

**Table 1** summarizes the baseline characteristics of the 89 patients included in the study. All patients received surgery plus chemotherapy and radiation therapy. The median age was 59 years (range, 32–78), 69 men (77.5%) and 20 women (22.5%). More than two thirds of the patients were in the stage T4 (69.7%) and more than half of the patients were in the stage N3.

**Table 1:** Clinicopathological characteristics of the study cohort.

Characteristics	No. of patients (n = 89)
**Sex, n (%)**	
**Male**	69 (77.5%)
**Female**	20 (22.5%)
**Age, median year (range)**	59 (32–78)
**CEA**	
**Increase**	14 (15.7%)
**Normal**	71 (79.8%)
**Unknown**	4 (4.5%)
**T stage, n (%)**	
**pT1**	3 (3.4%)
**pT2**	5 (5.6%)
**pT3**	19 (21.3%)
**pT4**	62 (69.7%)
**N stage, n (%)**	
**pN1**	20 (22.5%)
**pN2**	19 (21.3%)
**pN3a**	31 (34.8%)
**pN3b**	19 (21.3%)
**LVI**	
**Positive**	42 (47.2%)
**Negative**	47 (52.8%)
**Baseline index**	
**NLR, median (range)**	2.12 (0.46–6.97)
**PLR, median (range)**	127.92 (39.62–443.14)
**LMR, median (range)**	4 (1.50–28.14)
**SII, median (range)**	440 (47.54–3150.71)
**SIRI, median (range)**	0.84 (0.06-3.95)
**PIV, median (range)**	182.35 (12.15–1825.13)
**PNI, median (range)**	46.5 (34.00–65.00)

LVI, lymphovascular invasion; NLR, neutrophil-to-lymphocyte ratio; PLR, platelet-to-lymphocyte ratio; LMR, lymphocyte-to-monocyte ratio; SII, systemic immune inflammation index; SIRI, systemic inflammation response index; PIV, pan-immune-inflammation value; PNI, prognostic nutrition index.

### The cut-off value of inflammatory markers

**Figure 1:**
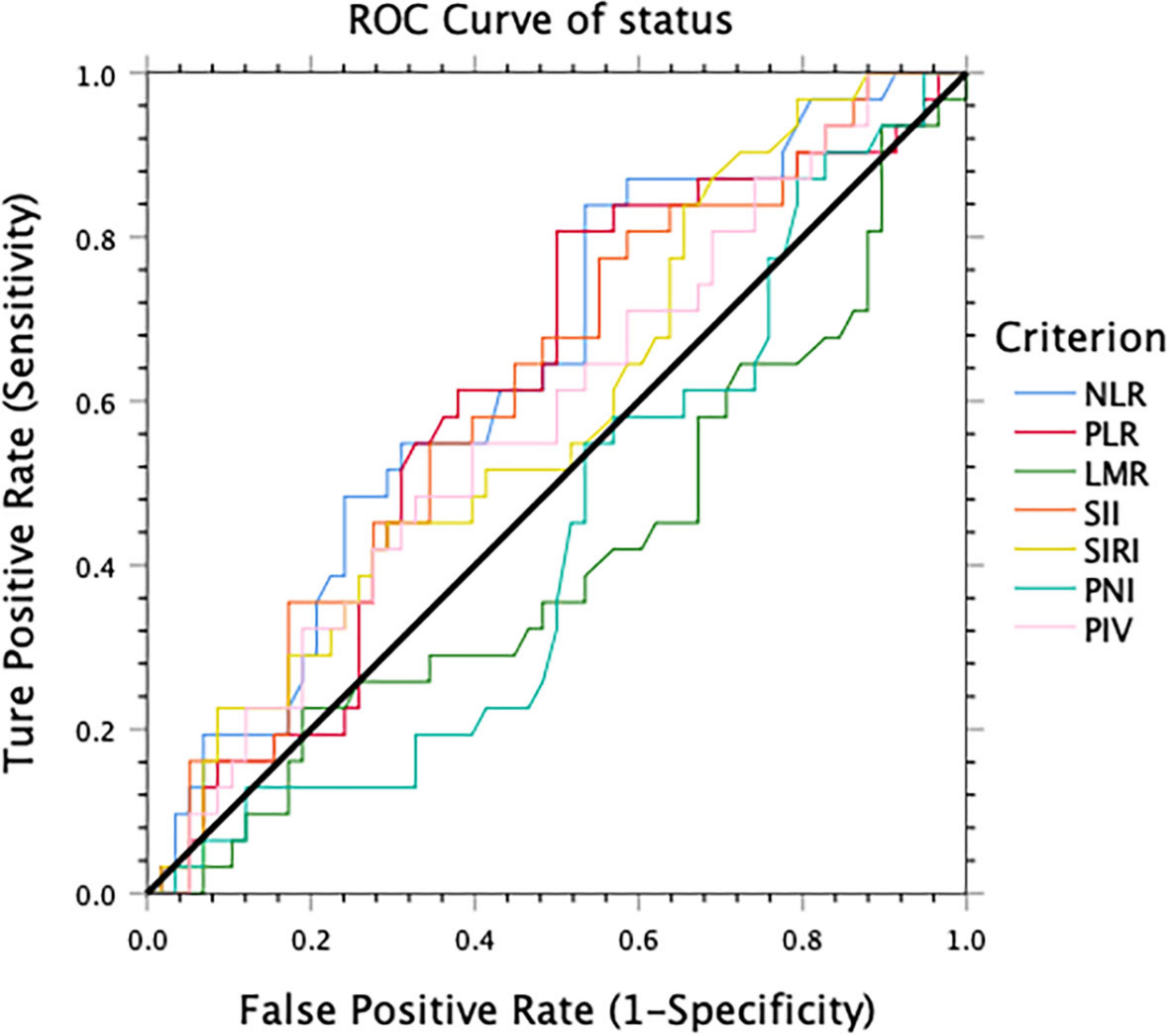
The optimal cut-off value for 3-year disease-free survival obtained using receiver operating characteristic analysis.

The optimal cutoff value of these inflammatory markers for the 3-year DFS was obtained using ROC curve analysis. The cut-off values for NLR, PLR, LMR, SII, SIRI, PIV, and PNI were 1.7, 109.3, 2.9, 369.2, 0.58, 218.7, 48, respectively (**Figure 1**).

### Relationship between clinicopathological factors and 3-year DFS

Univariate Cox regression analysis was performed using age, sex, stage T, stage N, LVI, CEA level, NLR, PLR, LMR, SII, SIRI, PIV, and PNI (**Table 2**). Univariate analysis showed that stage N (HR 2.656, *P =* 0.012), LVI (HR 2.621, *P =* 0.011), NLR (HR 3.642, *P =* 0.008), PLR (HR 3.137, *P =* 0.012), LMR (HR 0.406, *P =* 0.017), and PNI (HR 0.399, *P =* 0.033) were associated with the 3-year DFS of patients with LAGC treated with adjuvant chemoradiotherapy. There was no significant difference between individuals ≥65 years and those <65 years of age. Factors with *p*-value <0.05 in the univariate analysis were also included in the multivariate analysis. Univariate analysis showed that there was no statistically significant difference in the 3-year DFS among patients with different neutrophil counts or platelet counts. Multivariable Cox analysis (**Table 2**) indicated that the NLR (HR 2.991, *P =* 0.034) and pN3b (HR 2.463, *P =* 0.026) were independent predictors for DFS among gastric cancer patients treated with adjuvant chemoradiotherapy.

**Table 2:** Univariate and multivariate analysis for 3-year DFS in patients with gastric cancer treated with D2 gastrectomy and chemoradiotherapy.

Characteristics	n	Univariate		Multivariate	
		HR, 95%CI	*P*-value	HR, 95%CI	*P*-value
**Sex**					
**Male**	69	1		–	*-*
**Female**	20	0.548 (0.210–1.429)	*0.219*	–	*-*
**Age (years)**					
**<65**	69	1		–	*-*
**≥65**	20	1.397 (0.642–3.036)	*0.399*	–	*-*
**CEA**					
**Normal**	71	1		–	*-*
**Increase**	14	1.114 (0.426–2.911)	*0.826*	–	*-*
**T stage**					
**pT1-2**	7	1		–	*-*
**pT3-4**	82	23.893 (0.148–3845.444)	*0.221*	–	*-*
**N stage**					
**pN1-3a**	70	1		1	
**pN3b**	19	2.656 (1.240–5.689)	*0.012*	2.463 (1.117–5.434)	*0.026*
**LVI**					
**Negative**	47	1		1	
**Positive**	42	2.621 (1.251–5.491)	*0.011*	2.105 (0.975–4.548)	*0.058*
**Neutrophil count**					
**<2.0×10^9^/L**	9	1		–	*-*
**2.0-6.0×10^9^/L**	72	1.410 (0.334–5.957)	*0.640*	–	*-*
**>6.0×10^9^/L**	8	2.429 (0.444–13.296)	*0.306*	–	*-*
**Platelet count**					
**<150×10^9^/L**	10	1		–	*-*
**150-300×10^9^/L**	67	0.965 (0.291–3.200)	*0.954*	–	*-*
**>300×10^9^/L**	12	0.656 (0.132–3.256)	*0.606*	–	*-*
***NLR***					
**≤1.70**	32	1		1	
**>1.70**	57	3.642 (1.394–9.517)	*0.008*	2.991 (1.085–8.248)	*0.034*
***PLR***					
**≤109.3**	35	1		1	
**>109.3**	54	3.137 (1.286–7.654)	*0.012*	2.211 (0.827–5.910)	*0.114*
***LMR***					
**≤2.90**	20	1		1	
**>2.90**	69	0.406 (0.194–0.851)	*0.017*	0.702 (0.324–1.521)	*0.370*
***SII***					
**≤369.2**	33	1		–	*-*
**>369.2**	56	2.211 (0.951–5.138)	*0.065*	–	*-*
***SIRI***					
**≤0.58**	19	1		–	*-*
**>0.58**	70	3.002 (0.912–9.882)	*0.071*	–	*-*
***PIV***					
**≤218.7**	55	1		–	*-*
**>218.7**	34	1.611 (0.795–3.264)	*0.186*	–	*-*
***PNI***					
**≤48**	55	1		1	
**>48**	34	0.399 (0.172–0.927)	*0.033*	0.802 (0.316–2.036)	*0.642*

HR, hazard ratio; DFS, disease free survival; SIRI, systemic inflammation response index; NLR, neutrophil to lymphocyte ratio; LMR, lymphocyte to monocyte ratio; PLR, platelet to lymphocyte ratio; SII, systemic immune inflammation index; PIV, pan-immune-inflammation value; PNI, prognostic nutrition index.

### NLR and survival outcomes

**Figure 2:**
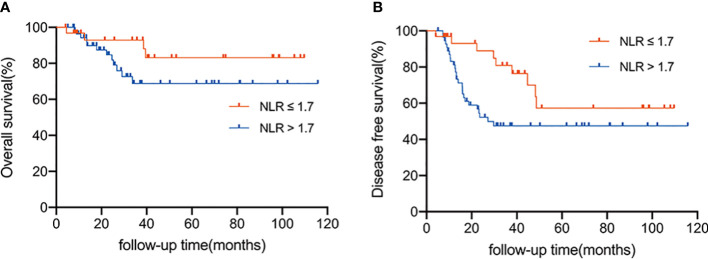
**(A)** Overall survival according to the NLR status (≤ 1.7 vs. > 1.7). **(B)** Disease-free survival according to the status of NLR status (≤ 1.7 vs. > 1.7).

With a median follow-up of 29.1 months (range 4.1 to 115.8 months), the 3-year OS was 78.9% and the 3-year DFS was 59.1%. Patients were divided into two groups according to the optimal cut-off value: the low NLR group (NLR 1.7) and the high NLR group (NLR>1.7). In total, 32 (36.0%) and 57 (64.0%) patients were divided into the low and high NLR groups, respectively. The survival rate of these two groups was significantly different (**Figures 2A**, **B**). The 3-year OS rate of the patients in the low NLR group and the high NLR group was 92.9% and 68.8%, respectively (*P =* 0.045); the 3-year DFS rate in the low NLR group and the high NLR group was 80.9% and 47.5%, respectively (*P =* 0.005). To identify a better model for predicting the outcome of patients, we also calculated the NLR-PLR score ranging from 0 to 2 as follows: score of 2, high NLR (>1.7) and high PLR (>109.3); score of 1, high NLR or high PLR; score of 0, neither high NLR nor high PLR. NLR-PLR scores of 0, 1, and 2 were observed in 18 (20.2%), 31 (34.8%), and 40 (44.9%) patients, respectively. However, overall survival differences according to the NLR-PLR score were not significant (*P >* 0.05).

### Role of NLR in initial failure patterns

**Figure 3:**
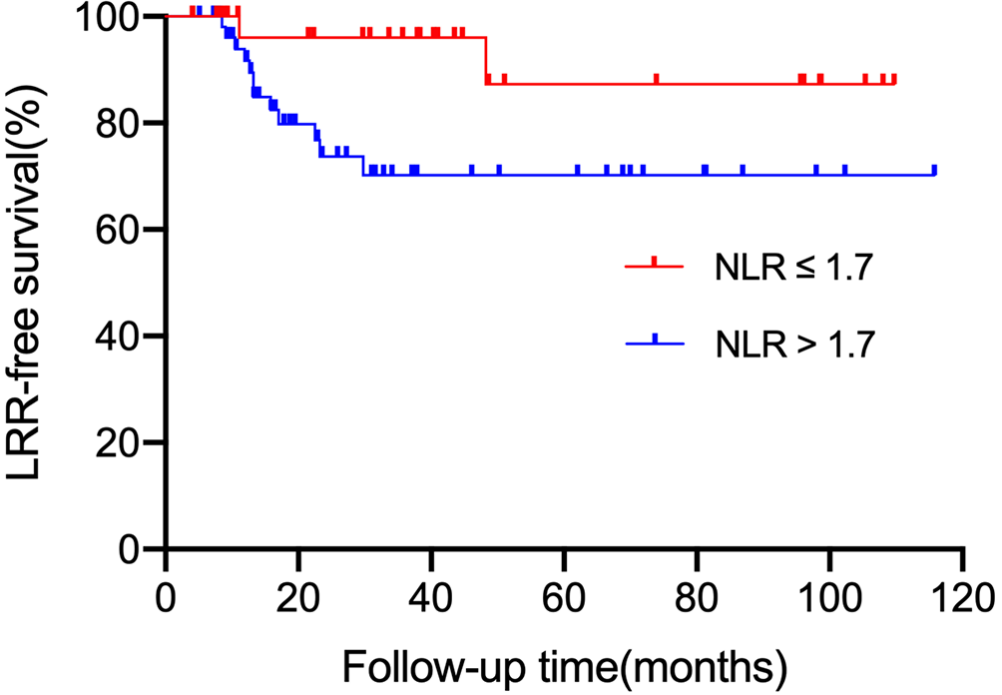
LRR-free survival according to NLR status (≤ 1.7 vs. > 1.7). *LRR*, local regional recurrence.

We also analyzed the first site of recurrence, LRR was more common in the high NLR group. The LRR-free survival rate of patients in the low NLR group was much higher than those in the high NLR group (96.0% vs. 70.2%, *P =* 0.017, **Figure 3**). The aortic lymph node was the most common site of LRR (7/14). But no patient in the low NLR group experienced para-aortic lymph node recurrence. In contrast, the distant or peritoneal metastasis rate was not significantly different between the high NLR group and the low NLR group (*P =* 0.066, 0.117, respectively).

## Discussion

Despite the large number of clinical trials, it remains uncertain whether patients can benefit from chemoradiotherapy after D2 dissection for gastric cancer. Many studies have shown that systemic inflammatory and nutritional biomarkers are independent predictors of various malignancies, including gastric cancer (15, 29–35). Furthermore, several inflammatory indicators, such as the NLR (13, 36), PLR (36), nutritional index (37), and LMR (38) have also been reported as potential prognostic indices for chemoradiotherapy. However, it remains to be determined whether inflammatory indicators can predict the prognosis of gastric cancer after D2 dissection with chemoradiotherapy. To our knowledge, this is the first study to define the prognostic value of peripheral blood inflammation indices in gastric cancer after D2 dissection treated with chemoradiotherapy. Our study found that N stage (pN3b) and NLR were independent prognostic factors for DFS in patients with LAGC after D2 dissection treated with chemoradiotherapy. In our previous study, we showed that pN3b is an independent adverse prognostic factor in gastric cancer after D2 dissection (24). Thus, in this study, we focused mainly on the correlation between NLR and the prognosis of patients with LAGC after D2 gastrectomy.

Several researchers have demonstrated a close relationship between NLR and tumor prognosis in various malignancies, including gastric cancer (10–13). Similar to previous studies (11, 39), our current research also showed that patients with high NLR have relatively shorter OS and DFS than patients with low NLR. Furthermore, our study found that the baseline NLR value was strongly associated with gastric cancer LRR after D2 dissection and chemoradiotherapy. Before this study, several retrospective studies explored the prognostic role of NLR in patients with gastric cancer undergoing radiation therapy and chemotherapy. The multicenter study by Meral et al. (39) showed that NLR was significantly correlated with lymph node status and prognosis in gastric carcinoma. Subsequently, Tetsushi et al. (36) found that baseline NLR was closely related to the clinical outcomes of gastric cancer after chemotherapy or chemoradiotherapy. Recently, Liu et al. (40) also found that NLR could be considered a routine potential prognostic factor for gastric cancer after surgery. According to our study, baseline NLR could be a new prognostic factor for disease progression and survival in patients with LAGC after D2 dissection and chemoradiotherapy.

There are several possible explanations for the relationship between increased NLR and poor tumor prognosis. Increased expression of tumor-related inflammatory mediators and cytokines, such as tumor necrosis factor-α, interleukin-1 (IL-1), and IL-6, have been reported to be increased in gastric cancer cases and other cancers (41). These inflammatory mediators may cause neutrophilia and lymphocytopenia, leading to a higher NLR (42). The host immune response to cancer is lymphocyte-dependent. Conversely, neutrophils are reported to be the main source of circulating chemokines and cytokines, and are major contributors to tumor-related angiogenesis (43). Meanwhile, consistent with our findings, patients with an elevated NLR can experience a relatively poor oncologic outcome and need more intensive treatment.

There is no generally recognized cut-off value for these indexes; some studies have selected the median value of each inflammatory and nutritional index as the cut-off level, and others have set the cut-off value based on existing literature. In previous published studies, the NLR cutoff values ranged from 2–6 (13, 36, 39). In this study, we draw ROC curves to determine the best cut-off value. A cutoff value of 1.7 for NLR could be optimal for LAGC patients treated with adjuvant chemoradiotherapy after D2 dissection. According to this cut-off value, patients were divided into two groups: low NLR group (NLR ≤1.7) and high NLR group (NLR >1.7).

However, there are several inherent limitations to our study. First, our study was a small retrospective study based on a single center, which may have potential selection bias. Another limitation was the short follow-up time.

In conclusion, the present study confirmed that baseline NLR could serve as a valuable independent prognostic factor for LAGC patients after D2 dissection who receive adjuvant chemoradiotherapy. However, large prospective studies with long-term follow-up should be performed to confirm our findings.

## Data availability statement

The raw data supporting the conclusions of this article will be made available by the authors, without undue reservation.

## Ethics Statement

This study was approved by the Institutional Medicine Review Board and a waiver for patient consent was obtained.

## Author contributions

S-BW conducted data extraction, quality appraisal, data synthesis and analysis, and drafted the manuscript. W-GC designed the protocol, performed the search, data extraction, quality appraisal, data synthesis and interpretation, and drafted the manuscript. J-YC and CX contributed to writing and editing the manuscript. LC and RC determined the scope of the review and contributed to protocol design and writing and editing the manuscript. GC had full access to the data, takes responsibility for data integrity, and is the guarantor of the review. All authors contributed to the article and approved the submitted version.

## Funding

This study was supported in part by the National Natural Science Foundation of China (grant numbers 81803164).

## Conflict of interest

The authors declare that the research was conducted in the absence of any commercial or financial relationships that could be construed as a potential conflict of interest.

## Publisher’s note

All claims expressed in this article are solely those of the authors and do not necessarily represent those of their affiliated organizations, or those of the publisher, the editors and the reviewers. Any product that may be evaluated in this article, or claim that may be made by its manufacturer, is not guaranteed or endorsed by the publisher.
